# Functional Characterization and Expression Analyses Show Differential Roles of Maternal and Zygotic Dgcr8 in Early Embryonic Development

**DOI:** 10.3389/fgene.2020.00299

**Published:** 2020-03-31

**Authors:** Zeyao Zhu, Yun Liu, Wen Xu, Taian Liu, Yuxin Xie, Kathy W. Y. Sham, Ou Sha, Christopher H. K. Cheng

**Affiliations:** ^1^Department of Anatomy, Histology and Developmental Biology, School of Basic Medical Sciences, Shenzhen University, Shenzhen, China; ^2^School of Biomedical Sciences, The Chinese University of Hong Kong, Hong Kong, China; ^3^State Key Laboratory of Biocontrol, Institute of Aquatic Economic Animals, Sun Yat-sen University, Guangzhou, China; ^4^Shenzhen Institutes of Advanced Technology, Chinese Academy of Sciences, Shenzhen, China

**Keywords:** maternal dgcr8, zygotic dgcr8, microRNAs, zebrafish, embryonic development

## Abstract

Dgcr8 is involved in the biogenesis of canonical miRNAs to process pri-miRNA into pre-miRNA. Previous studies have provided evidence that Dgcr8 plays an essential role in different biological processes. However, the function of maternal and zygotic Dgcr8 in early embryonic development remains largely unknown. Recently, we have reported a novel approach for generating germline-specific deletions in zebrafish. This germline knockout model offers an opportunity to investigate into the differential roles of maternal or zygotic Dgcr8. Although germline specific dgcr8 deletion has no influence on gonad development, maternal or zygotic dgcr8 is essential for embryonic development in the offspring. Both maternal *dgcr8* (M*dgcr8*) and maternal zygotic *dgcr8* (MZ*dgcr8*) mutants display multiple developmental defects and die within 1 week. Moreover, MZ*dcgr8* mutant displays more severe morphogenesis defects. However, when a miR-430 duplex (the most abundantly expressed miRNA in early embryonic stage) is used to rescue the maternal mutant phenotype, the Mdgcr8 embryos could be rescued successfully and grow into adulthood and achieve sexual maturation, whereas the MZ*dgcr8* embryos are only partially rescued and they all die within 1 week. The differential phenotypes between the Mdgcr8 and MZ*dgcr8* embryos provide us with an opportunity to study the roles of individual miRNAs during early development.

## Introduction

The model organism zebrafish (*Danio rerio*) is an excellent system for studying the developmental process of organogenesis. However, its application is limited due to the lack of a conditional knockout (cKO) platform. Recently, we have developed a novel approach for achieving cKO in zebrafish (Liu et al., 2017), making it possible to study the maternal genes in specific tissues or cells. Using this platform, maternal gene knockout zebrafish can be constructed by specifically deleting maternal genes in the oocytes. Furthermore, maternal mutant models cannot be constructed using the usual knockout methods because of the lethality of maternal homozygous gene knockouts. Therefore, our approach makes it possible to study the function of maternal factors during embryonic development in zebrafish.

Generally, maternal factors are important components in early embryonic development during the cleavage stage to maintain normal meiosis prior to the activation of the embryonic genome (Dosch et al., 2004; Wagner et al., 2004; Harvey et al., 2013). In zebrafish, the first maternal-effect mutant called *janus* was discovered, and this mutant appears to have a phenotype with a partially penetrant axis duplication, but the mechanism of this phenotypic change was not known at that time (Abdelilah et al., 1994). Subsequently, numerous maternal effect genes were discovered in the 1990s (Haffter et al., 1996; Kane et al., 1996; Gritsman et al., 1999; Miller-Bertaglio et al., 1999; Sirotkin et al., 2000). In mammals, thanks to the application of gene knock-out technology in mice, many studies have demonstrated that mammalian embryogenesis also needs maternal regulation, and numerous maternal factors have been identified in mice (Christians et al., 2000; Tong et al., 2000; Payer et al., 2003; Wu et al., 2003; Wagner et al., 2004).

Usually, maternal mutants have no influence on the mother, but normal zygotic development is seriously disrupted even if the mutated allele fails to pass to the offspring (Marlow, 2010). In zebrafish, several maternal genes have been identified by mutagenesis screening (Solnica-Krezel et al., 1996; Dosch et al., 2004; Wagner et al., 2004). Studies of the maternal mutants have revealed that maternal factors not only promote early embryonic cell division but also direct cell fate and organize the embryonic body plan (Tadros and Lipshitz, 2009; Marlow, 2010).

In the present study, we have used a number of approaches to examine the difference in M*dgcr8* and MZ*dgcr8* after rescuing. The absence of maternal Dgcr8 resulted in severe defects, including the disruption of gastrulation or epiboly movement, brain malformation, hematopoiesis defects, heart defects, and body curvature changes. Our findings also demonstrate that most of these defects could be rescued except hematopoiesis and heart defects in MZ*dgcr8*. Further analysis demonstrated that miR-430 partially rescued hematopoiesis and heart function, suggesting that a multitude of miRNAs including miR-430 are the key factors in maintaining development of the heart and hematopoiesis system in early embryonic development.

## Materials and Methods

### Generation of M*dgcr8* and MZ*dgcr8* Zebrafish

We obtained M*dgcr8* and MZ*dgcr8* mutant zebrafish using kop: Cre-UTR-nanos3, *dgcr8* cKO females crossed with wild-type male and kop: Cre-UTR-nanos3, *dgcr8* cKO male, respectively. M*dgcr8* and MZ*dgcr8* embryos were collected from natural spawning in the zebrafish facility system with a 14 h/10 h light/dark cycle, and embryos were collected and kept in 28.5°C incubators with a light/dark cycle. The developmental staging of the embryos was classified according to the universal principle (Kimmel et al., 1995).

### RNA Isolation and Real-Time PCR

Total RNA samples were isolated from zebrafish embryos at several developmental stages (shield, 75%-epiboly, prim-6, prim-16) with an RNeasy Mini Kit (Qiagen). The amount and purity of the RNA samples were determined by NanoDrop 2000 spectrophotometry (Thermo Fisher Scientific). The cDNA was synthesized using the PrimeScript RT Reagent Kit (Takara). Real-time PCR was performed on an ABI PRISM 7900 Sequence Detection System (Applied Biosystems) using the SYBR Green I Kit (Applied Biosystem). The primers used in this study are listed in Supplementary Table S1. The mRNA transcript levels were normalized against the ef14α transcript level.

### Whole-Mount *in situ* Hybridization

Whole mount *in situ* hybridization (ISH) was performed as described (Li et al., 2014). cDNA fragments were amplified by RT-PCR with specific primers (Supplementary Table S1), followed by *in vitro* transcription with either T7 RNA polymerase to generate the antisense probe using the DIG RNA Labeling Kit (Roche, United States). Images were captured using a SZX16 stereomicroscope with fluorescence imaging (Olympus, Japan).

### Morphological and Histological Analysis

After anesthetization and dissection of adult zebrafish, gonads including testes and ovaries were carefully obtained and then transferred into a culture dish containing 60% L-15 medium for investigation. After fixed in Bouin’s fixative buffer (Sigma, United States) or 4% PFA (Sigma, United States) overnight at 4°C, the gonad samples were dehydrated and embedded in paraffin, and then sectioned at 5 μm thickness on a Leica microtome. After drying overnight, the slides were stained with hematoxylin and eosin (H&E) according to the standard protocol (Sabaliauskas et al., 2006). Folliculogenesis (Wang and Ge, 2004) and spermatogenesis (Leal et al., 2009) were staged accordingly.

### Mitotic Centrosome Detection

Embryos were fixed with 4% PFA overnight at 4°C and dehydrated in absolute methanol for at least 20 min at −20°C. After washing thrice with 1 × PBST, the embryos were then permeabilized in −20°C acetone for 8 min. After washing, the embryos were blocked with blocking buffer (2% lamb serum, 0.1% dimethyl sulfoxide, 0.1% bovine serum albumin, and 0.2% Triton-X100 in PBS) for 1 h at room temperature. The embryos were treated with anti-γ-tubulin antibody (Sigma) at a dilution of 1:1000 in blocking buffer overnight at 4°C. After washing with PBST for 30 min three to five times at room temperature, the embryos were incubated in fluorescein-conjugated secondary goat antirabbit antibody (Alexa-fluor 555, 1:500, Life Technology, United States) at 1:1000 dilution for 2 h at room temperature. The embryos were then washed eight times in PBST for 5 min each. The whole embryos were mounted in a confocal dish with DAPI Fluoromount-G medium (Southern Biotech) and incubated for 5 min prior to imaging.

### Phosphorylation of Histone H3 (PH3) Antibody Staining

Zebrafish embryos were first fixed at 4°C in 4% PFA overnight. After washing three times with PBST (PBS + 0.2% Triton-X100) for 5 min each, the embryos were permeabilized for 8 min in −20°C acetone, washed in PBST three times, blocked for 30 min at room temperature with blocking buffer (2% lamb serum, 0.1% dimethyl sulfoxide, 0.1% bovine serum albumin, and 0.2% Triton-X100 in PBS), and then incubated overnight at 4°C in polyclonal rabbit anti-phospho-histone H3 antibody (Cell Signaling Technology, United States) at a concentration of 1:1000. After washing five times with PBST for 5 min each, the embryos were incubated for 2 h at room temperature in secondary goat antirabbit antibody (Alexa-fluor 555, 1:500, Life Technology, United States). Then, the embryos were washed eight times with PBST for 5 min each and processed for imaging. Changes in the numbers of mitotic cell were quantified by counting the number of phospho-histone 3 (PH3) positive cells in the whole body of the embryo taking the mean of three embryos.

### Rescue of M*dgcr8* and MZ*dgcr8* Using miR-430 Duplex

To perform the rescue of the M*dgcr8* and MZ*dgcr8* phenotypes, the miR-430 duplex mimics were synthesized by Shanghai GenePharma Co., Ltd. Working solutions were prepared in RNase free water at 20 μM and stored at −20°C. For the rescue, 1–2 nl of miR-430 duplexes (a mixture of miR-430a, miR-430b, and miR-430c at a ratio of 1:1:1) were injected into one-cell stage M*dgcr8* and MZ*dgcr8* embryos. The phenotypic changes in embryo development were recorded on a stereomicroscope.

### Quantifying Heart Rate

From the digital video of 52 h post fertilization (hpf) or 4 dpf embryos, the number of heartbeats were counted for 20 s. Heart rate (beats/min) was calculated by multiplying the number of beats counted by three. Four embryos were counted per group.

### Touch Sensitivity Assay

At approximately 48 hpf of development, wild-type, M*dgcr8* and MZ*dgcr8* mutant embryos were touched at the trunk region adjacent to the yolk extension with a glass needle (Giraldez et al., 2005). The response was recorded for three consecutive stimuli and assessed by a touch response behavioral assay (Granato et al., 1996). No response was determined based on the absence of tail movement after touching the embryo three times. A weak response was determined as a wiggle of the tail after stimulation. A strong response was defined as when the fish tail gave a c-bend opposite to the site of the touch. The whole touch procedure was captured on digital video. Different groups of embryos were assessed twice.

### O-Dianisidine Staining

O-dianisidine solution was prepared at 0.7 mg/ml in 100% ethanol and protected from exposure to light. The working stain solution was prepared by mixing 1 ml of 0.7 mg/ml O-dianisidine, 1 ml of ddH_2_O, 250 μl of 100 mM sodium acetate, and 50 μl of 30% hydrogen peroxide (Giraldez et al., 2005). After anesthetization, the embryos were transferred into a 24-well plate and 0.5 ml of the working solution was added. Embryos were incubated in the dark at room temperature for 20 min, washed three times with PBS, and fixed with 4% PFA overnight. Embryos were placed in 100% glycerol for imaging. The images were acquired on a SZX16 stereomicroscope microscope (Olympus, Japan).

### mRNA Sequencing and Small RNAs Sequencing

For mRNA and small RNA sequencing, total RNA was extracted from MZ*dcgr8* and control embryos at 30% epiboly stage of early development representing 4.5 hpf using miRNeasy Mini Kit (Qiagen). RNA integrity was assessed on an Agilent Bioanalyzer 2100 (Agilent). Each group was consisted of 50 embryos. Qualified total RNA was further purified by RNA Clean XP Kit (Beckman) and RNase-Free DNase Set (Qiagen). Library construction and sequencing was performed by Shanghai Biotechnology Corporation. The 30% epiboly stage was chosen because it is a key period in MZT during early vertebrate development. At this stage, the embryo starts to shift from utilization of maternal mRNAs and factors, and initiates zygotic transcription.

### Statistical Analysis

In this study, all raw data were analyzed by the GraphPad Instat software (GraphPad Software, United States). Mean values ± SEM. *P* < 0.05 were considered statistically significant using one-way ANOVA. Tukey test was used for multiple comparisons to determine statistical differences. All experiments were performed at least three times to confirm reproducibility.

## Results

### Germline Specific Dgcr8 Deletion Has No Functional Influence on Gonad Development

**FIGURE 1:**
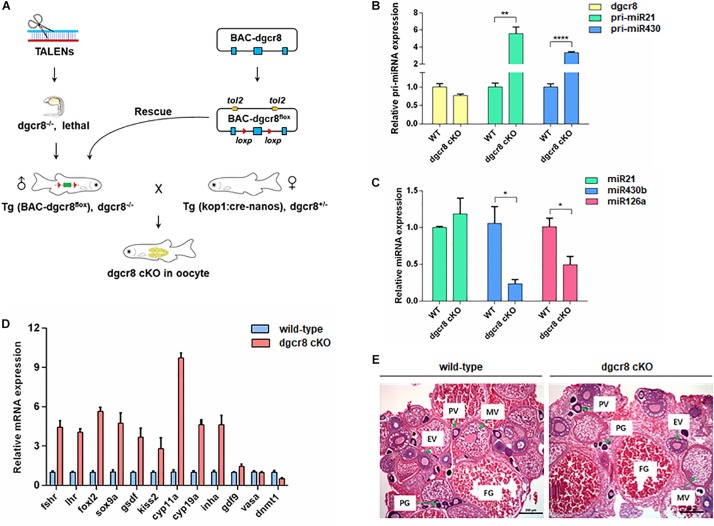
Germline specific dgcr8 deletion has no functional influence on gonad development. **(A)** A basic schematic diagram of generating *dgcr8* germline conditional knockout zebrafish. **(B)** Real-time analysis of the expression of *dgcr8* mRNA and primary miRNAs in ovary of *dgcr8 kop*-cKO fish. **(C)** Real-time PCR analysis of the expression of mature miRNAs in ovary of *dgcr8 kop*-cKO fish. **(D)** Expression of germline markers in the ovary of *dgcr8 kop*-cKO. **(E)** Histology of ovaries in the wild-type and *dgcr8 kop*-cKO female zebrafish. *p < 0.05, **p < 0.01, and ****p < 0.0001.

As shown in the schematic diagram in Figure 1A, we obtained germline specific dgcr*8* cKO zebrafish using Tg(BAC-*dgcr8*^*flox*^), *dgcr8^–/–^* male crossed with Tg (kop: cre-UTR-nanos3, CMV: EGFP), *dgcr8*^±^ female. To determine the expression levels of *dgcr8* and individual miRNAs in adult ovary of the germline specific *dgcr8* cKO, qRT-PCR was employed, and the results suggested that *dgcr8* was not significantly downregulated, but primary miRNAs were upregulated significantly due to the absence of dgcr8 processing in the adult ovary (Figure 1B), and mature miRNAs were downregulated sharply because of disruption of the miRNA biogenesis pathway (Figure 1C). The majority of the germline markers were upregulated in the ovary of *dgcr8* cKO except vasa and dnmt1 (Figure 1D). The *dgcr8* cKO ovary morphology was also validated by histology, with all the ovarian follicle stages found in the *dgcr8* cKO ovary with no obvious difference compared with the wild-type ovarian follicles (Figure 1E).

### MZdcgr8 Embryos Display More Severe Morphogenesis Defects Than Mdcgr8

**FIGURE 2:**
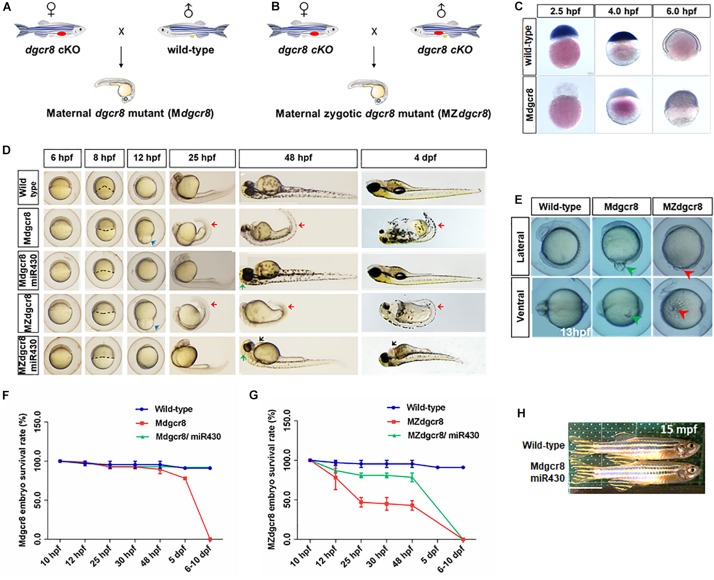
Rescue of M*dgcr8* and MZ*dgcr8* using *miR-430* duplex. **(A)** A schematic diagram of generating M*dgcr8* mutant zebrafish by out-crossing with wild-type male. **(B)** A schematic diagram of generating MZ*dgcr8* mutant zebrafish by in-crossing. **(C)** The expression of *dgcr8* was analyzed by *in situ* hybridization in early embryonic stage. **(D)** M*dgcr8* and MZ*dgcr8* exhibit gastrulation defect, brain malformations, body curvature, and heart developmental defects compared with wild-type. *miR-430* duplex was used to rescue the defects. **(E)** Epiboly defects in M*dgcr8* and MZ*dgcr8.* At 14 hpf, the phenotype of yolk excision could be observed in MZ*dgcr8* mutant (5 X), but not in M*dgcr8*. **(F)** M*dgcr8* embryonic survival rate become normal after rescue. **(G)** MZ*dgcr8* mutant embryos only survived up to 6–10 dpf after rescue. **(H)** M*dgcr8* mutant embryos after rescue could develop to adults and exhibit proper sex ratio. Scale bar 1 cm.

To further determine the necessity of maternal Dgcr8 in the early developmental process, we obtained a fish line with maternal Dgcr8 deletion (M*dgcr8* and MZ*dgcr8*) by crossing *dgcr8* cKO female fish with wild-type male fish (Figure 2A) and *dgcr8* cKO male fish (Figure 2B). M*dgcr8* could provide direct evidence for the role of maternal factors because of the presence of zygotic Dgcr8 of paternal origin in the early developmental stage. The ISH signal of *dgcr8* transcripts decreased from 2.5 to 6 hpf in the wild-type embryos (Figure 2C), but the signal in the M*dgcr8* embryos increased due to the presence of zygotic *dgcr8* (Figure 2C). Real time-PCR analysis showed that dgcr8 transcripts were decreased during the developmental process from the 256-cell stage to the 75% epiboly stage in the MZ*dgcr8* embryos (Supplementary Figure S1).

From the shield stage onward, M*dgcr8* and MZ*dgcr8* mutant embryos exhibited development delay and developed more slowly than the wild-type embryos by approximately 3–4 h at 25 hpf (Figure 2D). Their epiboly movements were disrupted with a longer animal-vegetal axis but a shorter dorsal-ventral axis, and the epiboly level was decreased significantly compared to the wild-type embryos at 12 hpf (Figure 2D). Intriguingly, the part of the yolk was excised to induce the next developmental stage in MZ*dgcr8* mutants. The accumulation of cells in the region of the anterior axial mesendoderm showed a reduced extent at 13 hpf, but yolk excision did not happen in the M*dgcr8* mutant (Figure 2E), suggesting that zygotic *dgcr8* might partially make up for the epiboly defects. From 25 hpf, MZ*dgcr8* and M*dgcr8* mutant embryos exhibited tail curvature until death on 6–10 dpf (Figure 2D).

### miR-430 Duplex Successfully Rescues M*dgcr8* but Not MZ*dgcr8*

In zebrafish, the miR-430 family is the most abundant miRNA family during early embryogenesis (Chen et al., 2005). During the maternal to zygotic transition (MZT), miR-430 is responsible for targeting and clearing more than 200 mRNAs in zebrafish embryos (Giraldez et al., 2006). In this study, we found that the M*dcgr8* mutant could be saved and they could even survive to adulthood after rescue, but MZ*dgcr8* could only be partially rescued and they died on 6–10 dpf (Figure 2D). At 8 hpf, the epiboly level of M*dgcr8* and MZ*dgcr8* decreased compared with the wild-type, but significantly improved after rescue (Figure 2D). In particular, the epiboly defects showing a longer animal-vegetal axis and a shorter dorsal-ventral axis (Figure 2D, blue arrows) were rescued completely by miR-430 duplex at 12 hpf. Furthermore, the body curvature (Figure 2D, red arrows) was rescued successfully in M*dgcr8* and MZ*dgcr8* at 25 and 48 hpf. However, miR-430 could rescue M*dgcr8* heart defects but failed to do so in the MZ*dgcr8* embryos and hydropericardium (hp) was observed clearly in the MZ*dgcr8/*miR-430 group at 48 hpf (Figure 2D, black arrows). Moreover, the brain malformation was rescued successfully in Mdgcr8 and MZdgcr8 embryos at 48 hpf (Figure 2D).

After rescue, the M*dgcr8* embryonic survival rate became normal compared to the wild-type (Figure 2F), but no MZ*dgcr8*/miR-430 embryo survived beyond 10 dpf (Figure 2G), indicating that *dgcr8* or miRNAs other than miR-430 were necessary for the later developmental processes. At 15 months post fertilization (mpf), the morphology of the M*dgcr8*/miR-430 mutants were normal and exhibited a proper sex ratio (male: female = 12:6) (Figure 2H), and they could also produce offspring normally by in-cross. Taken together, the miR-430 duplex could rescue M*dgcr8* embryos completely but not MZ*dgcr8*.

### Maternal Dgcr8 Regulates Mitosis During Early Embryonic Development

**FIGURE 3:**
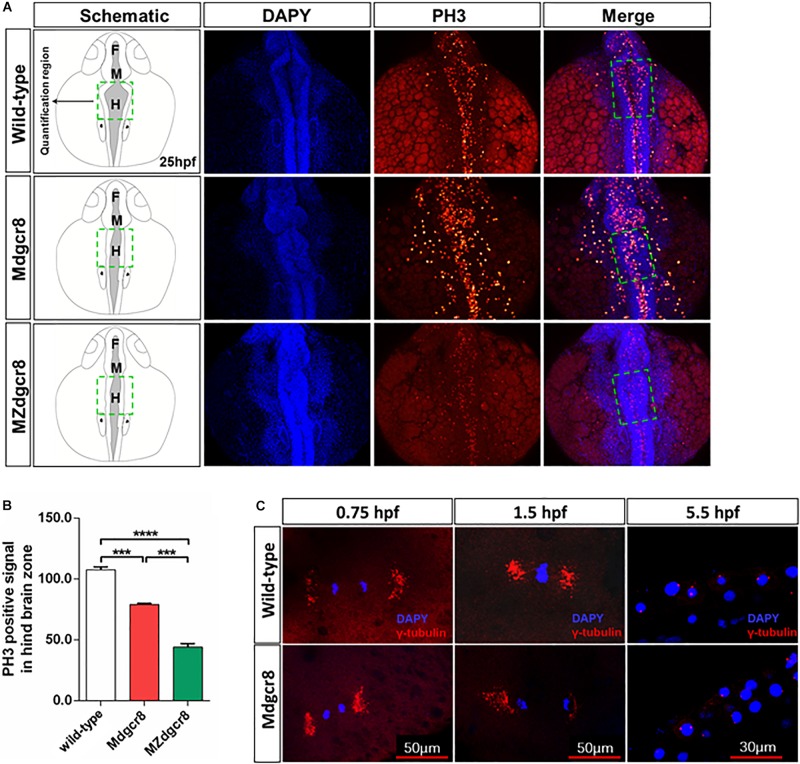
Maternal Dgcr8 regulates mitosis during early embryonic development in M*dgcr8* and MZ*dgcr8*. **(A)** Representative images of 25 hpf WT, M*dgcr8* and MZ*dgcr8* embryos showing the distribution of PH3 immunolabeling cells **(B)** and quantification of positive cells in the hindbrain before the otic vesicle in the dashed box region (*N* = 3). **(C)** Mitotic centrosomes were detected using γ-tubulin immunostaining. ***p < 0.001 and ****p < 0.0001.

To investigate the developmental delay in M*dgcr8* mutant embryos, proliferation was assayed by a PH3 immunolabeling G2/M phase mitosis marker. At 25 hpf, the hindbrain zone of the M*dgcr8* embryos showed significant decreases in the PH3 immunolabeling of the positive cells compared to wild-type embryos, and MZ*dgcr8* exhibited a significant decrease in comparison with M*dgcr8* (Figures 3A,B). Moreover, the body of M*dgcr8* and MZ*dgcr8* were smaller than the wild-type embryos (Figure 3A). To further examine the organization of maternal mutant mitotic centrosomes, γ-tubulin immunostaining was used. Compared with wild-type, M*dgcr8* centrosomes appeared normal at 0.75, 1.5, and 5.5 hpf during early developmental stage (Figure 3C). These results suggested that maternal Dgcr8 might regulate cell proliferation through spindle organization other than centrosome organization.

### Loss of Maternal or Zygotic *Dgcr8* Causes Cardiac Defects

In zebrafish, the cardiac progenitor cells are localized in the anterior lateral plate mesoderm as two parts on both sides of the embryo at the 5-somite stage (∼12 hpf). Then the two parts of the cardiac progenitor cells migrate toward the middle-line and combine together at the 18-somite stage (∼18 hpf). Next they reorganize to constitute a primitive heart tube that starts peristaltic contraction at the 26-somite stage (∼22 hpf), and finally develop into two cardiac chambers, namely, the atrium and ventricle, which become more organized at the long-pec stage (∼48 hpf) (Huang et al., 2009).

**FIGURE 4:**
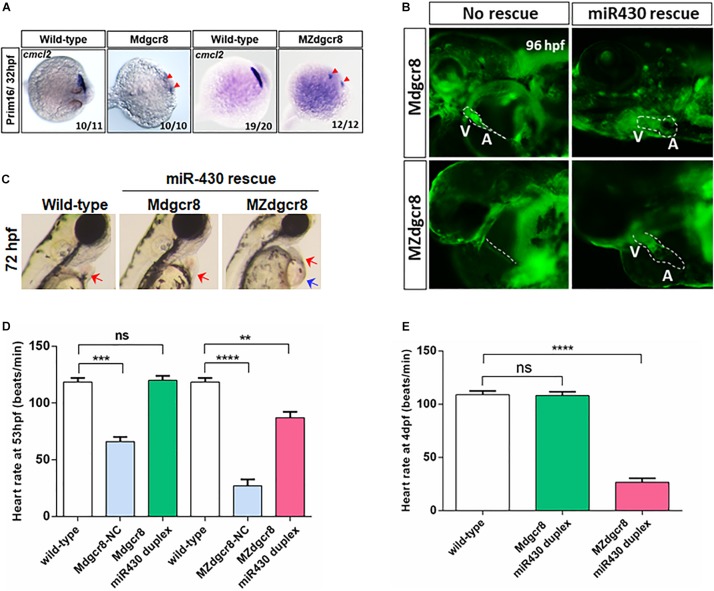
Heart defects was successfully rescued in M*dgcr8*, but not in MZ*dgcr8*. **(A)** The cardiac progenitor cells failed to migrate to the middle-line at prim-16 stage (32 hpf) in M*dgcr8* and MZ*dgcr8* (red arrows). **(B)** After miR430 rescue, cardiogenesis appeared normal including structure of atrium and ventricle. **(C)** MZ*dgcr8* failed to be rescued by mir430, exhibiting hydropericardium phenotype. **(D,E)** Heart-rate results indicated that the heart defects were completely rescued in M*dgcr8*, but only partially in MZ*dgcr8*, and MZ*dgcr8* heart rate gradually decreased at 4 days. **p < 0.01, ***p < 0.001, and ****p < 0.0001.

To investigate the process of cardiac development, we used ISH to detect the cardiac progenitor cells and found that these cells (marked by *cmcl2*) were specified but failed to migrate toward the middle-line at the Prim-16 stage (32 hpf) in the M*dgcr8* and MZ *dgcr8* mutant, but wild-type embryos formed a complete heart tube (Figure 4A). In terms of morphology, the rescued M*dgcr8* exhibited a normal cardiac structure compared to the wild-type (Figures 4B,C), but the rescued MZ*dgcr8* only had a thinner cardiac tube and developed a hp syndrome (blue arrow) at 72 hpf (Figures 4B,C).

To determine whether maternal Dgcr8 participates in heart development or cardiac function, we investigated the heart rate of the embryos. M*dgcr8* and MZ*dgcr8* had significantly decreased heart rates compared to the wild-type, but no significant changes were observed in the rescued M*dgcr8*, suggesting that miR-430 duplex successfully rescued the heart defects (Figure 4D). The mean heart rate in MZ*dgcr8* embryos was only 26 beats/min and this could reach up to 87 beats/min after rescue, which was still much lower than the wild-type (118 beats/min), indicating that the heart defects could be rescued partially by miR-430 in MZ*dgcr8* (Figure 4D). At 4 dpf, the heart rate of the rescued M*dgcr8* was normal, but the heart rate of the rescued MZ*dgcr8* decreased gradually because of body malformations (Figure 4E). It could thus be concluded that both maternal and zygotic *dgcr8* are essential for heart development and cardiac function.

### Loss of Maternal or Zygotic *Dgcr8* Leads to Hematopoietic Defects

In zebrafish, hematopoietic development is divided into two waves. The first wave, named the primitive wave, mainly encompasses induction of erythrocytes and myeloid cells. Primitive blood cells begin to circulate throughout the body of the embryo at 24 hpf (Orkin and Zon, 2008; Paik and Zon, 2010). The second wave is the definitive wave, which occurs in later development and mainly produces the hematopoietic stem and progenitor cells (HSPCs) and multipotent progenitors. Subsequently, HSPCs differentiate into all of the mature blood cells to maintain the embryos throughout life (Ellett and Lieschke, 2010).

**FIGURE 5:**
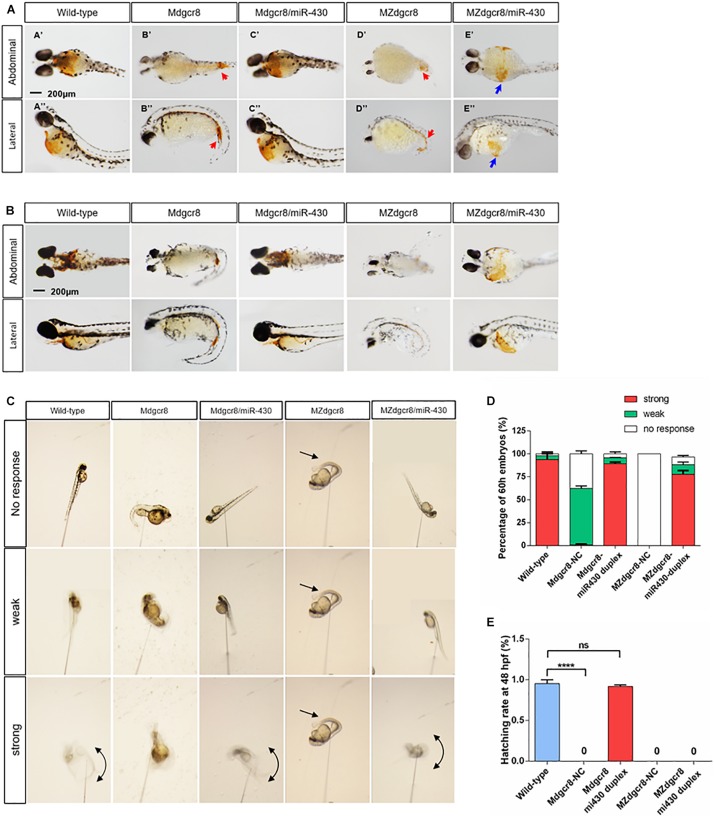
Maternal mutants exhibit reduced erythropoiesis and could be rescued in M*dgcr8*, but not in MZ*dgcr8*. Whole embryo O-dianisidine staining assay was used for assessing functional hemoglobin in mature primitive erythrocytes in wild-type, M*dgcr8*, MZ*dgcr8*, and *miR-430* rescued groups at 50 **(A)** and 74 hpf **(B)**. Hemoglobinized cells accumulate in the yolk sac in wild-type but mainly accumulate in the caudal vein (red arrows) in MZ*dgcr8* and MZ*dgcr8* mutants. 10–15 embryos per groups. Scale bar: 200 μm. **(C)** The touch response was categorized as strong, weak, and no response based on the analysis of the video frames. **(D)** At 60 hpf, the rescue rate was determined in wild-type and maternal mutant embryos. **(E)** At 48 hpf, the hatching rate was detected in wild-type and maternal mutant embryos. ****p < 0.0001.

To analyze functional hemoglobin in mature primitive erythrocytes in rescued embryos, we employed O-dianisidine staining and found that no O-dianisidine-positive erythroid cells were observed in the yolk sac, but staining accumulation was observed in the tail caudal vein of the maternal mutants at 50 hpf (Figures 5A–B’,B”D’,D”, red arrows). These phenotypes were consistent with qualitative visual observation of a slower circulation and decreased heart rate in Dgcr8 maternal mutant embryos. *Dgcr8* maternal mutants displayed a developmental delay that became more severe as the embryos grew older, and O-dianisidine staining was observed at 74 hpf but with no improvement in hemoglobin function (Figure 5B). Moreover, the level of O-dianisidine staining in the 74 hpf mutant embryos was still lower than that of the wild-type at 48 hpf, indicating that the decrease in O-dianisidine staining was unlikely due to the developmental delay alone. After rescuing by the miR-430 duplex, M*dgcr8* could be rescued completely but was only partially rescued in MZ*dgcr8* (Figures 5A–C’,C”,E’,E”), indicating that the hematopoiesis defects were successfully rescued in M*dgcr8* but not in MZ*dgcr8*.

### Touch-Induced Response Decreases in M*dgcr8* and MZ*dgcr8*

At approximately 2 dpf, the wild-type larvae hatch and start swimming under external stimulation. Swimming occurs by increasing the fin beat amplitude and rhythmic left-right axial cycle response of the tail (Granato et al., 1996). To investigate the role of Dgcr8 in neuronal development, a touch sensitivity assay was employed to determine the level of the escape response. At approximately 60 hpf, wild-type embryos responded to mechanosensory stimulation with a contralateral movement of the tail against where the stimulus was applied, and the tail then exhibited a characteristic c-bend (Granato et al., 1996). The touch response was then categorized as strong, weak, or no response. Analysis of the video frames showed that embryos were almost motionless and failed to respond to touch in M*dgcr8* (no response, 37.65%, *n* = 15) and MZ*dgcr8* (no response, 100%, *n* = 23); or generated a little twitch of the tail in M*dgcr8* (weak, 61%, *n* = 25) and MZ*dgcr8* (weak, 0%) (Figures 5C,D). Only 0.9% of the M*dgcr8* embryos displayed a strong response of the tail upon stimulation, and no MZ*dgcr8* embryos showed a strong movement (Figures 5C,D). After rescuing by the miR-430 duplex, the level of touch response improved significantly in both M*dgcr8* (strong, 89%, *n* = 14) and MZ*dgcr8* (strong, 77%, *n* = 15) embryos (Figures 5C,D). Moreover, the hatching rate of embryos is related to the level of spontaneous coiling movement inside the chorion in addition to the secreted proteolytic enzymes required for chorion-softening. M*dgcr8* and MZ*dgcr8* failed to hatch from the chorion naturally and no coiling movements were observed. Interestingly, the hatch rate of M*dgcr8* could be rescued completely but not the hatch rate of MZ*dgcr8* (Figure 5E). These results revealed essential roles of Dgcr8 during zebrafish neurogenesis.

### Expression Changes of Downstream Genes in miRNA Biogenesis Pathway

In M*dgcr8* mutant embryos, maternal *dgcr8* mRNA could not be detected in early developmental stage (Supplementary Figure S1), the expression of other genes downstream of the canonical miRNA biogenesis pathway (*drosha*, *dicer*1, *xpo5*, and *ago2*) was significantly upregulated at the 256-cell and sphere stage (Supplementary Figure S1). This might be the cumulative outcome of ablation of pre-miRNAs due to the lack of maternal Dgcr8. Surprisingly, such a cumulative phenotype was not found in the MZ*dgcr8* mutant (Supplementary Figure S1). The expression level of pri-miR-430 was nearly sixfold that of the wild-type at the sphere stage (Supplementary Figure S1). Pri-miR-430 in M*dgcr8* embryos was also accumulated abundantly due to the lack of Dgcr8 processing.

Similarly, *dgcr8* mRNA was also not detected in the MZ*dgcr8* mutant embryos (Supplementary Figure S2), but the expression level of other genes downstream of the canonical miRNA biogenesis pathway was normal (Supplementary Figure S2). These results indicated that all of the miRNA biogenesis transcripts were of maternal origin and were decreased during the developmental process. However, the primary miRNAs were not of maternal origin, and they might begin to be transcribed during the MZT period. Pri-miR-430 was only detected in the sphere stage (Supplementary Figure S2), while expression of pri-miR-21 peaked in the shield stage (Supplementary Figure S2). Both pri-miR-430 and pri-miR-21 in MZ*dgcr8* embryos accumulated abundantly due to the lack of Dgcr8 processing compared to the wild-type embryos (Supplementary Figure S2). In conclusion, these results showed that the canonical miRNA biogenesis pathway was disrupted because of Dgcr8 ablation in MZ*dgcr8* mutant embryos.

### Small RNA-Seq Analysis

We have also analyzed the early embryonic transcriptomes and small RNA at the 30% epiboly stage (4.5 hpf) using small RNA-seq MZ*dgcr8* embryos. A total of 13,846,020 raw reads and 12,087,801 raw reads were obtained for the wild-type and MZ*dgcr8* embryos, respectively. We finally obtained 13,759,185 clean effective reads in wild-type and 12,011,626 in MZ*dgcr8* after removal of the reads of the adaptor sequence and low-quality sequences, including those smaller than 18 nt in length and smaller than 10 D in base molecular weight. By blasting with the *Danio* miRbase library, 5830 annotated counts were obtained in the wild-type embryos, but only 591 counts were annotated in MZ*dgcr8* (Supplementary Figures S3A,B). In the wild-type embryos, small RNAs with lengths of 23 nt were detected in the largest ratio, followed by 21 and 22 nt (Supplementary Figure S3C), and this is consistent with the 21–23 nt range of mature miRNAs. However, small RNAs with 21–23 nt in length failed to be detected in MZ*dgcr8* embryos (Supplementary Figure S3C). From the sum value reads, we also know that small nucleolar RNA (snoRNA) was downregulated significantly (Supplementary Figure S3D), and this is consistent with a previous report that DGCR8 regulated snoRNA biogenesis (Macias et al., 2012). These data revealed that almost all the miRNAs failed to express due to the ablation of maternal and zygotic Dgcr8 in early embryos.

**FIGURE 6:**
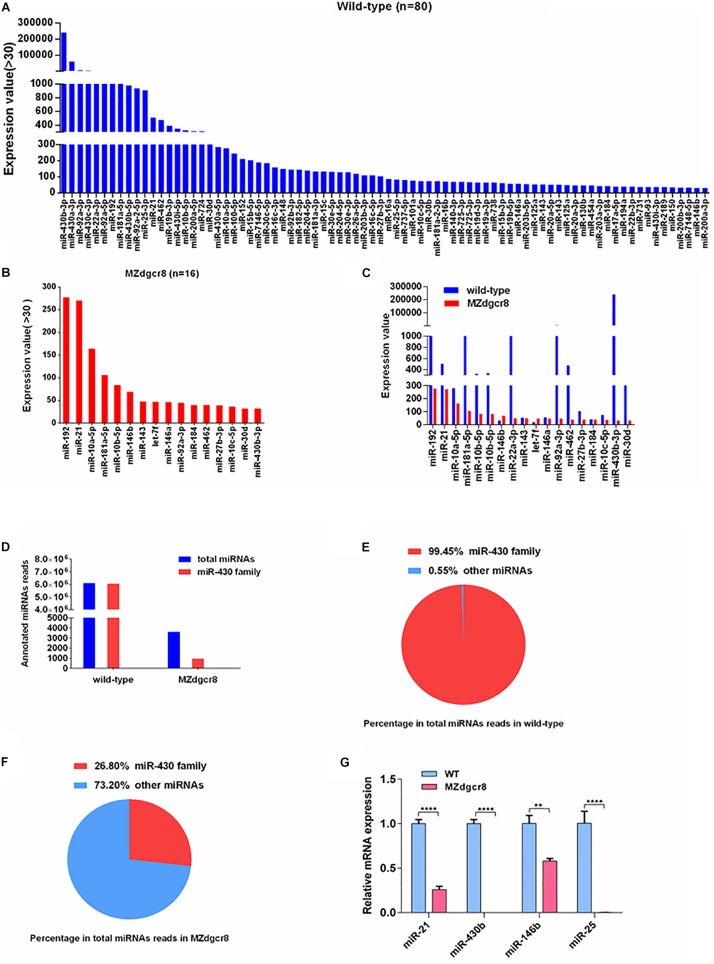
Expression value of miRNAs in wild-type and MZ*dgcr8* embryos and qPCR validations. **(A)** 80 miRNAs for values greater than 30 were obtained in wild-type embryos. **(B)** 16 miRNAs for values greater than 30 were obtained in MZ*dgcr8* embryos. **(C)** miRNAs with higher values in wild-type and MZ*dgcr8*. **(D)** Total miRNAs read count and *miR-430* family read count in wild-type and MZ*dgcr8*. **(E)** Percentage of *miR-430* family in total miRNAs read count in wild-type. **(F)** Percentage of *miR-430* family in total miRNAs read count in MZ*dgcr8*. **(G)** Validation of some mature miRNAs by real-time PCR. **p < 0.01 and ****p < 0.0001.

To investigate the expression level or variation of specific miRNAs in early embryonic development, miRNAs with expression values higher than 30 (reads count) were chosen for further analysis. We obtained 80 miRNAs in the wild-type embryos and only 16 miRNAs in MZ*dgcr8* for values greater than 30 (Figures 6A,B). In wild-type embryos, the miR-430b is the most abundant miRNA (reads = 240,430) (Figure 6A) and in MZ*dgcr8* embryos, the most abundant miRNA is *miR-192* (reads = 277) (Figure 6B). Although most of the abundant miRNAs were depleted, several miRNAs still displayed a high residual expression level in MZ*dgcr8* embryos, such as *miR-192* (reads = 277), *miR-21* (reads = 270), and *miR-10a-5p* (reads = 164) (Figure 6C). The residual expression of *miR-21* was also validated using real-time PCR in this study (Figure 1C) although pri-miR21 was up-regulated about fivefold (Figure 1B). The residual expression of *miR-21* was reported in the previous study in *dgcr8* knockout mice (Yi et al., 2009). These results also revealed that there might be an alternative processing pathway independent of Dgcr8 for these miRNAs.

The miR-430 family, the most abundant miRNAs family during early stages, accounted for 99.45% of all the annotated miRNA reads in wild-type embryos (Figures 6D,E). However, the miR-430 family was severely depleted in MZ*dgcr8* embryos (Figures 6D,F). These RNA-seq data suggested that the miR-430 family is the most important member in embryonic development. We have also validated the results of some individual mature miRNAs using real-time PCR (Figure 6G).

### mRNA-Seq Analysis

**FIGURE 7:**
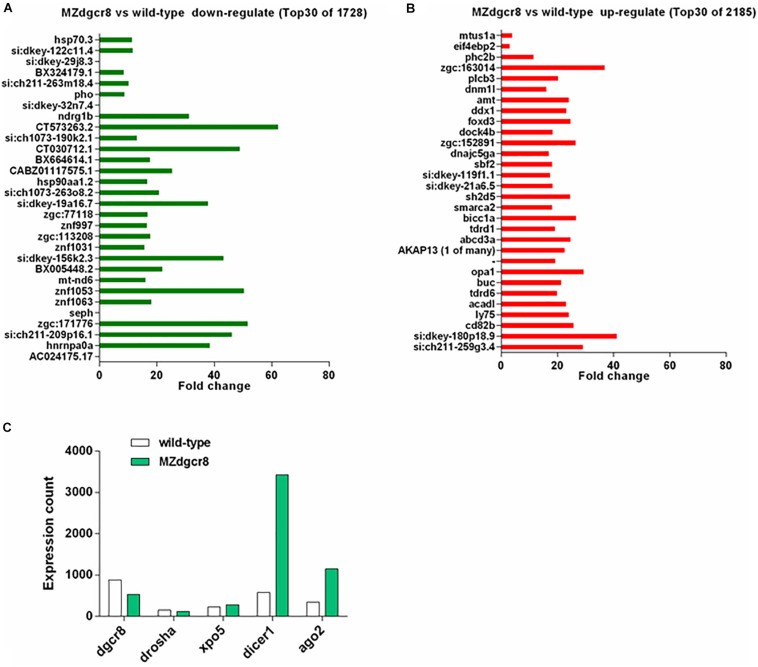
mRNA expression variation in wild-type and MZ*dgcr8* embryos during 30% epiboly stage. **(A)** Top 30 downregulated genes from RNA-seq data. **(B)** Top 30 upregulated genes from RNA-seq data. **(C)** Expression count of miRNAs biogenesis related genes.

In addition to miRNAs, we also analyzed the mRNAs using RNA sequencing. We found that 2185 genes were upregulated, and 1728 genes were downregulated during the 30% epiboly stage (Figures 7A,B). According to the fold change, the top 30 of the upregulated or downregulated genes were further analyzed. The nucleoid binding protein *hnrnpa0a* decreased more than 30-fold (Figure 7A), while *seph*, an essential gene regulating organ development in zebrafish, was absent in the MZ*dgcr8* embryo (Figure 7A). For the upregulated genes, the targets of miR-430 (*cd82b* and *gstm*) were significantly upregulated in MZ*dgcr8*, and this was verified using real-time PCR as described in our previous study (Liu et al., 2017). The *opa1* required for proper mitochondrial metabolism in early development was also upregulated. During the 30% epiboly stage, the Dgcr8 mRNA could be detected in MZ*dgcr8* embryos after deleting Exon 3 and Exon 4 of the *dgcr8* gene. The expression of other miRNA biogenesis genes including *xpo5*, *dicer*1, and *ago2* was also increased (Figure 7C).

To assess whether maternal Dgcr8 was directly related to different biological processes or some cellular components, Kyoto Encyclopedia of Genes and Genomes (KEGG) gene enrichment analysis was performed based on annotations of the zebrafish genes (GRCz10, ENSEMBL). Through KEGG pathway analysis, the scatter plot results displayed highly significant enrichment of genes belonging to “the protein processing in endoplasmic reticulum” (Supplementary Figure S4), with a *P-*value of 0.00075. The protein processing pathway was activated due to the upregulation of related genes, and this might be induced by the accumulation of mRNAs owing to the deficiency of Dgcr8 or miRNAs.

## Discussion

M*dgcr8* and MZ*dgcr8* mutants were generated using a BACK approach by germline specific deletion of Dgcr8 in zebrafish. Both M*dgcr8* and MZ*dgcr8* were malformed and finally causing embryo lethality. These observations indicate the essentiality of maternal Dgcr8 in early embryonic development. Previous studies observed that MZ*dicer* zebrafish exhibit developmental defects in gastrulation, brain development, and heart development (Giraldez et al., 2005). Compared with other miRNA biogenesis enzymes, Dgcr8 is the only member that specifically processes miRNAs because Dicer is also responsible for processing other small endogenous RNAs. Our data suggest that the canonical miRNAs and other small RNAs processed by Dicer play important roles in early development.

We found that most gene expression patterns were similar in M*dgcr8* and MZ*dgcr8* embryos. Compared with MZ*dcgr8*, the erythroid progenitors (marked by *gata1*) in M*dgcr8* were not severely affected at the 6-somite stage (12 hpf). Consistently, the erythroid cells were accumulated in the tail caudal vein of the M*dgcr8* embryos, but few erythroid cells were observed in MZ*dcgr8*. These results suggest that the specification of erythroid progenitors is regulated by zygotic Dgcr8 in addition to maternal Dgcr8.

The miR-430 family is the most abundant miRNA expressed during early embryonic development, accounting for 99.45% of all the annotated miRNA reads at the 30% epiboly stage using miRNA sequencing. Therefore, we have employed a miR-430 mimic to rescue the M*dgcr8* and MZ*dgcr8* mutants and found that M*dgcr8* mutants could be completely rescued by miR-430, but MZ*dgcr8* could only be partially rescued. A previous study observed that the developmental defects in MZ*dicer* mutants could be partially rescued by miR-430 (Giraldez et al., 2005), suggesting that miR-430 plays an important role in early development. However, rescue of the MZ*dgcr8* mutants by miR-430 is not sufficient for the MZ*dgcr8* mutant to survive to adulthood, indicating that other miRNAs processed by zygotic Dgcr8 are required for the later biological processes.

We further investigated the processes of neurogenesis, cardiogenesis, and hematopoiesis in the M*dgcr8* and MZ*dgcr8* mutants after miR-430 rescue. These results suggest that neurogenesis was completely rescued in both M*dgcr8* and MZ*dgcr8* mutants. Remarkably, brain morphogenesis was normal, and the touch response rate was not significantly changed in M*dgcr8* and MZ*dgcr8* mutants after rescue. It has been demonstrated that miR-430 could rescue brain morphogenesis successfully in MZ*dicer* mutants (Giraldez et al., 2005). These observations indicate that miR-430 is both necessary and sufficient for neurogenesis. In contrast, the defects in the development of the heart and hematopoiesis were not rescued by miR-430 in MZ*dgcr8*, consistent with previous study (Giraldez et al., 2005). It has been reported that miR-23 is essential for excessive endocardial cushion cell differentiation in zebrafish embryonic hearts (Lagendijk et al., 2011), and miR-218 mediates the formation of the linear heart tube in zebrafish during heart field migration (Fish et al., 2011). Studies have also demonstrated that miR-451 plays a crucial role in promoting erythroid maturation via its target transcript *gata2* in hematopoiesis (Pase et al., 2009; Cifuentes et al., 2010). These observations show that other individual miRNAs besides miR-430 participate in the process of cardiogenesis and hematopoiesis in early development.

To investigate the role of Dgcr8 in miRNA processing, we sequenced the small RNAs at the 30% epiboly stage in MZ*dgcr8* mutant embryos. Comparing mature miRNA reads between wild-type and MZ*dgcr8* mutants revealed a large decrease (by 90%) of miRNA reads in the MZ*dgcr8* mutants. Interestingly, although most of the microRNAs were depleted in the early embryonic stage, three miRNAs still displayed a high residual expression level in MZ*dgcr8* embryos, including miR-192, miR-21, and miR-10a-5p. These results revealed that these miRNAs might have alternative miRNA processing pathways independent of Dgcr8. A similar conclusion was reached in another study, suggesting that residual expression of miR-21 was detected in *Dgcr8* knockout mice (Yi et al., 2009).

## Conclusion

In summary, we have demonstrated that Dgcr8 or global miRNAs are essential for embryonic development. And miR-430 was sufficient to rescue the M*dgcr8* mutant but only partially rescued the MZ*dgcr8*. The MZ*dgcr8* rescue experiments provide us a platform for identifying the novel miRNA candidates in organogenesis, especially cardiogenesis and hematopoiesis.

## Data Availability Statement

GEO reference number: GSE146606 is the reference Series for our publication: https://www.ncbi.nlm.nih.gov/geo/query/acc.cgi?acc = GSE146606.

## Ethics Statement

All animal procedures were approved by the Animal Experimentation Ethics Committee of the Chinese University of Hong Kong and were performed according to the animal license issued and endorsed by Department of Health, the Government of the Hong Kong Special Administrative Region.

## Author Contributions

CC, ZZ, and YL conceived and designed the research. ZZ, YL, WX, TL, and YX developed the methods and performed the experiments. KS prepared the reagents. ZZ analyzed the data and wrote the manuscript. CC and OS edited the manuscript.

## Conflict of Interest

The authors declare that the research was conducted in the absence of any commercial or financial relationships that could be construed as a potential conflict of interest.
