# Supplementary material for: Functional Characterization and Expression Analyses Show Differential Roles of Maternal and Zygotic Dgcr8 in Early Embryonic Development
Source: Front Genet. 2020 Mar 31;11:299. doi: 10.3389/fgene.2020.00299 (PMC7136893; doi:10.3389/fgene.2020.00299)
Supplement: Supplementary file 1 [file Data_Sheet_1.PDF]

Supplementary Figures

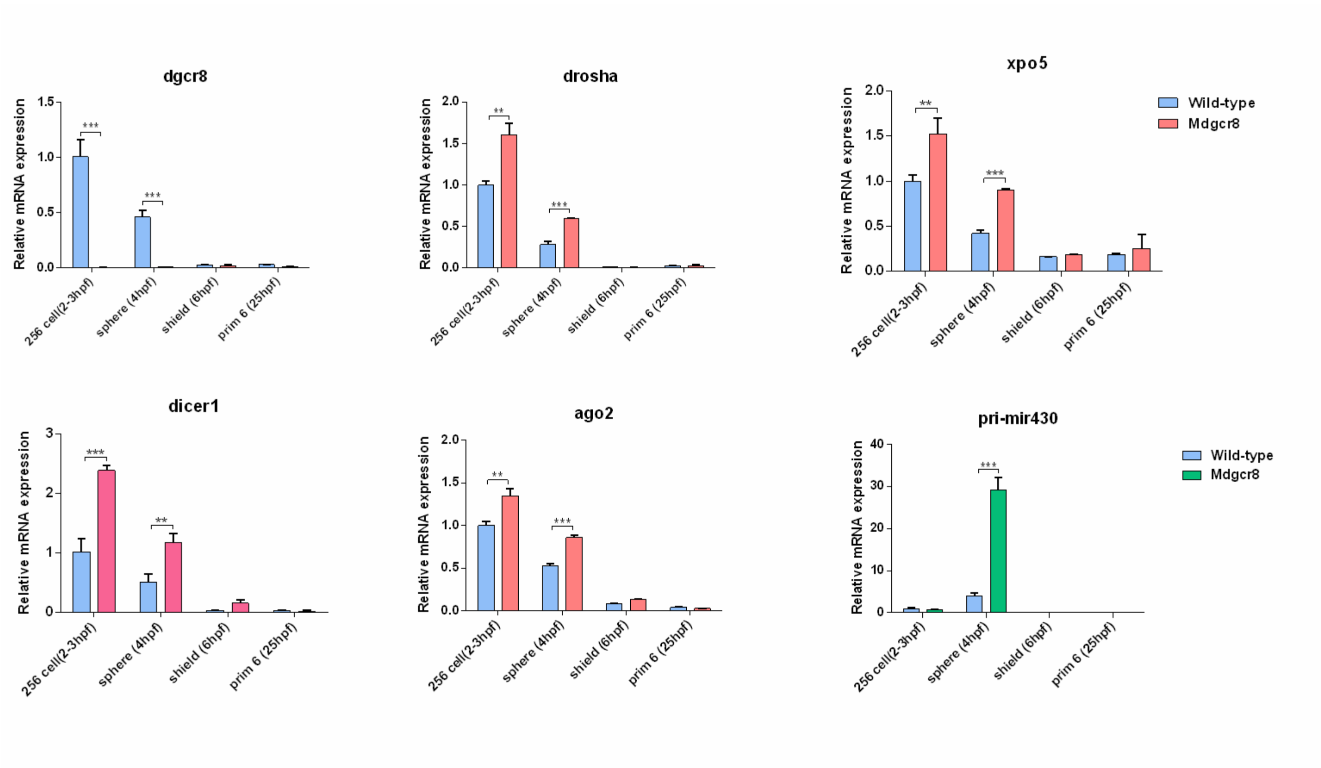

FIGURE S1 | Analysis of miRNAs biogenesis genes and primary miRNAs using real-time PCR in M*dgcr8*. Genes of canonical miRNA biogenesis pathway were chosen, including *dgcr8*, drosha, exo5, *dicer*1, and ago2. Real-time PCR analysis of pri-*miR-430* expression in embryos from the wild-type and M*dgcr8* mutant line in the 4 different stages.

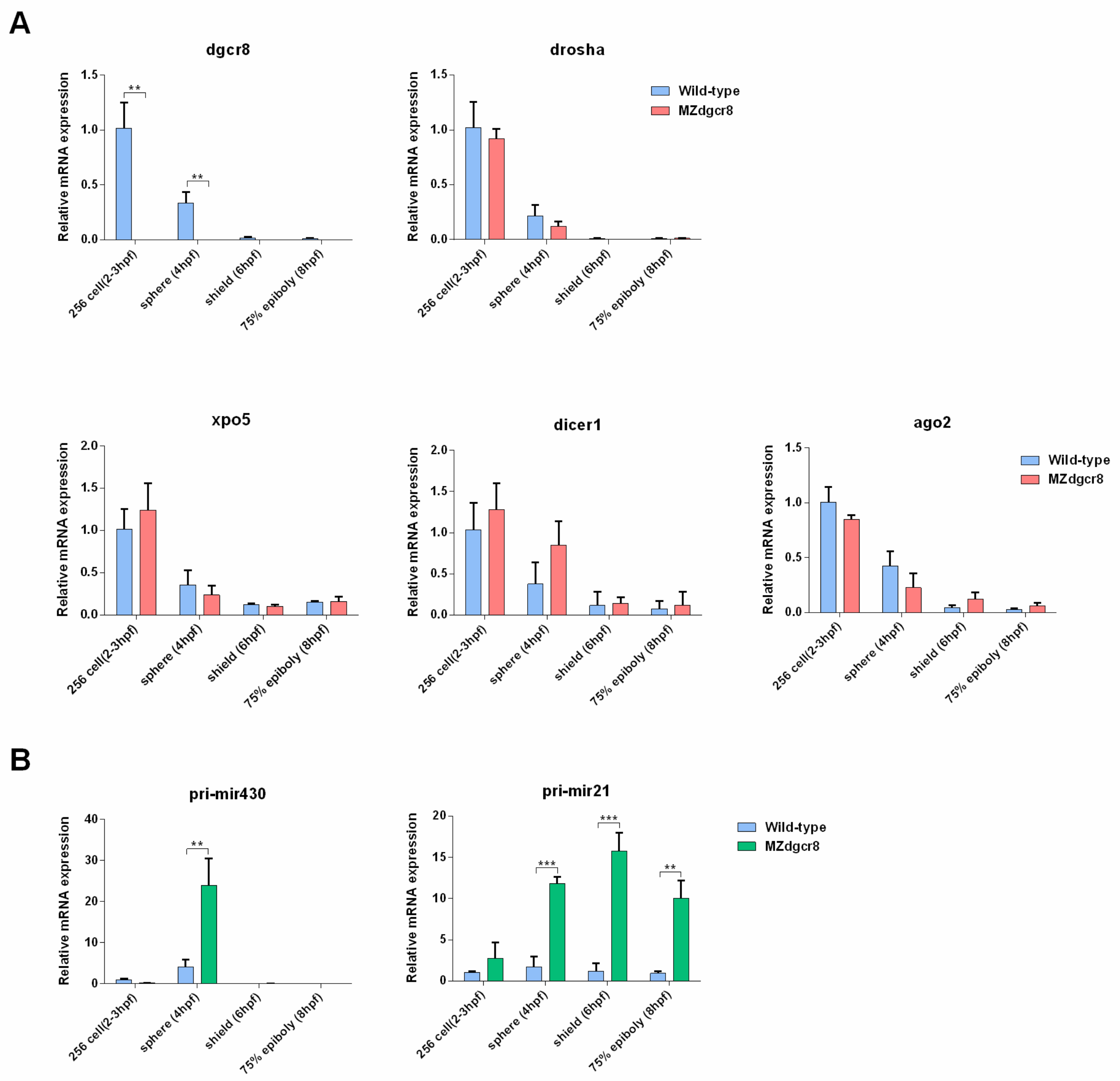

FIGURE S2 | Analysis of miRNAs biogenesis genes and primary miRNAs using real-time PCR in MZ*dgcr8*. **(A)** Genes of canonical miRNA biogenesis pathway were chosen, including *dgcr8, drosha, exo5, dicer1,* and *ago2*. **(B)** Realtime-PCR analysis of pri-miRNA expression in embryos from the wild-type and MZ*dgcr8* mutant line in the 4 different stages.

A

Name	Small RNAs	Annotated	Percentage	Ambiguously annotated
Wild.fq_trim_adaptor Small RNA sample	2,425,604	5,830	0.2%	4,208

Name	Percentage	Reads	Annotated	Percentage
Wild.fq_trim_adaptor Small RNA sample	0.2%	13,435,360	6,102,347	45.4%

Name	Ambiguously annotated	Percentage
Wild.fq_trim_adaptor Small RNA sample	6,081,888	45.3%

B

Name	Small RNAs	Annotated	Percentage	Ambiguously annotated
MZd8. fq_trim_adaptor Small RNA sample	2,882,961	591	0.0%	292

Name	Percentage	Reads	Annotated	Percentage
MZd8. fq_trim_adaptor Small RNA sample	0.0%	11,590,913	3,619	0.0%

Name	Ambiguously annotated	Percentage
MZd8. fq_trim_adaptor Small RNA sample	2,384	0.0%

D

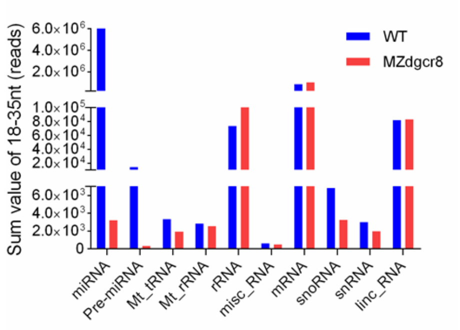

C

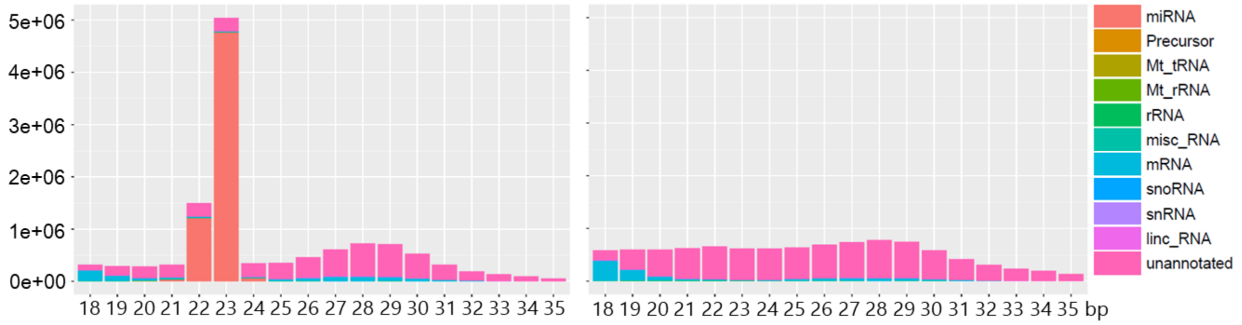

FIGURE S3 | An overview of the small RNA transcriptome analysis from wild-type and MZ*dgcr8* embryos in 30% epiboly stage. **(A)** Summary of small RNA mapping to the zebrafish genome in wild-type **(B)** Summary of small RNA mapping to the zebrafish genome in MZ*dgcr8*. **(C)** Length distribution of the sequences in wild-type embryos and MZ*dgcr8* embryos. **(D)** Sum reads value of small RNA with 18nt-35nt.

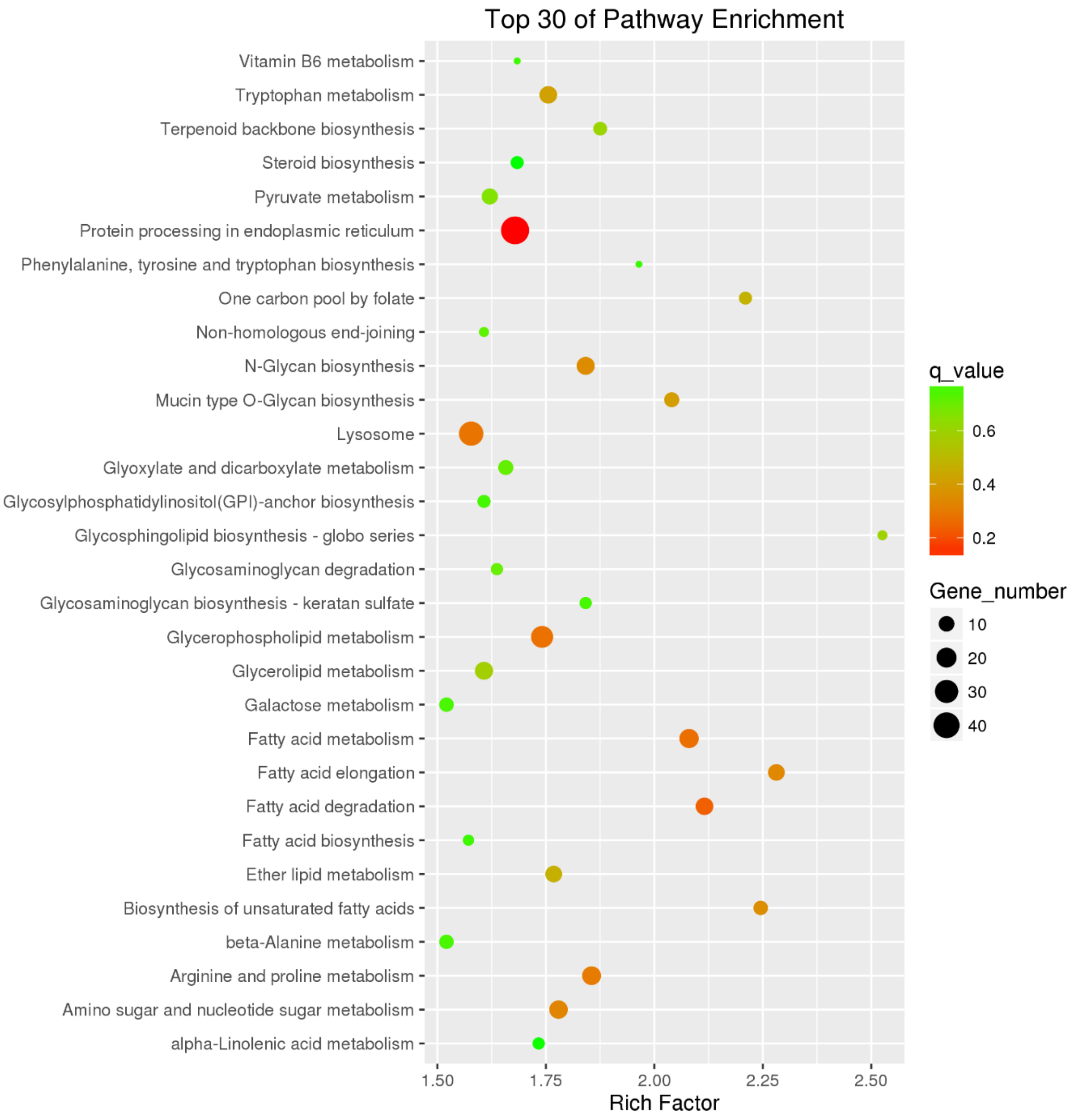

FIGURE S4 | Scatter plot of KEGG enrichment analysis (MZ*dgcr8* vs wild-type). Top 30 of pathway enrichment using KEGG enrichment analyses. Protein processing in endoplasmic reticulum (the largest red dot) represents the most affected pathway in the MZ*dgcr8* embryos. The greater the rich factor, the higher the degree of enrichment. The color of the dot represents q value, and the size of the dot represents the number of differentially expressed genes (DEGs) mapped to the reference pathways.

Supplementary table

TABLE S1 | List of oligos used in this study.

Primer name	Sequence (5′ → 3′)	Purpose
*dgcr8*_F0	CTTGAGTCAGACTCGAGAGGAG	Genotyping of global *dgcr8* knockout
*dgcr8*_R0	CTCACTGGAGCGAGCCGATGAG	
Cre-F1	GAACCTGATGGACATGTTCAGG	For PCR excision assay genotyping positive *Cre* transgenic fish
Cre-R1	AGTGCGTTCGAACGCTAGAGCCTGT	
dgcr8-cKO-F1	CAATAGGCACATAAAGAGGGGCTTAT AG	For *dgcr8*-cKO positive founder screening
dgcr8-cKO-R1	ATAAGAGAATAGAGATGAGGAGCGT GA	
dgcr8-cKO-F2	GCATGTGGCCACAAGGCCACACAAA GTGC	
dgcr8-cKO-R2	GTAGTGCAGGCAGGGAATGCTACTG G	
*dgcr8*_T7F	ACAGTACGTTAATACGACTCACTATA G GGCCCAATTGTGTTCTTGTATCAG	Amplification of zebrafish *dgcr8* cDNA containing open reading frame
*dgcr8*_T7R	GTGCACTCTCAGGTGCAGAAGTG	
pri-miR-21F	TTTCAGCCCCACCCTCTCCTCT	Realtime-PCR amplification of zebrafish Pri-miR-21
pri-miR-21R	GCTATCTGACACACTGGGAAAG	
pri-miR-25F	GCTACGCTACACTGATGCTACGC	Realtime-PCR amplification of zebrafish Pri-miR-25
pri-miR-25R	TCTTCTTCTGGTGAATGGAGGGG	
pri-*miR-430*F	AGTAACATGGGGACACTCCTTT	Realtime-PCR amplification of zebrafish Pri-*miR-430*
pri-*miR-430*R	CCCCAACTTGATAGCACTTTCT	
ef1α-qPCR-F	TGGAGGCCAGCTCAAACAT	Realtime-PCR amplification of zebrafish *ef1α*
ef1α- qPCR-R	ATCAAGAAGAGTAGTACCGCTAGCAT TAC	
dgcr8- RT-F1	ATCACCATTCAGACGGGGAG	Realtime-PCR amplification of zebrafish *dgcr8*
dgcr8- RT-R1	TTCCCGGTGGAGGTAGACTG	
*dicer*1-RT-F1	GCAAATTTTCCCTCGCCGTT	Realtime-PCR amplification of zebrafish *dicer1*
*dicer*1- RT-R1	GTCTTGCTTTCCTGCCTCCT	

drosha-RT-F1	CGACTATGACCGAGGACGTG	Realtime-PCR amplification of zebrafish *drosha*
drosha- RT-R1	GCGAGCGTGTTTACGATGTC	
ago2-RT-F1	CGCATGTGTTACGTGGCATC	Realtime-PCR amplification of zebrafish *ago2*
ago2- RT-R1	AGGAGACGCGGGAGCA	
Expotin5-RT-F1	CAGCCAAACATACCGACTGG	Realtime-PCR amplification of zebrafish *exportin5*
Expotin5-RT-R1	TGCTCCAGTATTTGCAGTCCA	
dre-*miR-430*a-duplex	uaagugcuauuuguuggguag	For M*dgcr8* and MZ*dgcr8* mutant rescue
dre-*miR-430*b-duplex	aaagugcuaucaaguugggguag	
dre-*miR-430*c-duplex	uaagugcuucucuuuggguag	
